# Similarity Theory of Withdrawn Water Temperature Experiment

**DOI:** 10.1155/2015/429781

**Published:** 2015-05-03

**Authors:** Yunpeng Han, Xueping Gao

**Affiliations:** State Key Laboratory of Hydraulic Engineering Simulation and Safety, Tianjin University, Tianjin 300072, China

## Abstract

Selective withdrawal from a thermal stratified reservoir has been widely utilized in managing reservoir water withdrawal. Besides theoretical analysis and numerical simulation, model test was also necessary in studying the temperature of withdrawn water. However, information on the similarity theory of the withdrawn water temperature model remains lacking. Considering flow features of selective withdrawal, the similarity theory of the withdrawn water temperature model was analyzed theoretically based on the modification of governing equations, the Boussinesq approximation, and some simplifications. The similarity conditions between the model and the prototype were suggested. The conversion of withdrawn water temperature between the model and the prototype was proposed. Meanwhile, the fundamental theory of temperature distribution conversion was firstly proposed, which could significantly improve the experiment efficiency when the basic temperature of the model was different from the prototype. Based on the similarity theory, an experiment was performed on the withdrawn water temperature which was verified by numerical method.

## 1. Introduction

Most reservoirs with more than 6 m depth are typically temperature-stratified as a result of surface heating and wind mixing. Water with low temperature withdrawn from deep reservoir is not suitable for irrigation and may easily bring hazard to aquatic organisms living downstream. Thus, water from different depths should be released (i.e., temperature), which is the concept of selective withdrawal.

Selective withdrawal has been studied for more than 40 years, and, in recent years, numerical methods are widely employed. A number of 1D, 2D, or even 3D numerical models were developed to predict the thermal structure or the withdrawn water temperature [1–4]. Some hydraulic analyses and extended theories of selective withdrawal were proposed. Jurka and Katavola examined the effect of intake dimensions and the finite thickness of the two-layer interface on the critical withdrawal conditions [5]. Wood extended the theory of selective withdrawal for both 2D and 3D flows and provided a relation for calculating the discharge from a two-layer flow when both layers are withdrawn [6]. Experimental study was also adopted by some researchers. Harleman and Elder studied two-layer flow via two kinds of skimmer wall intake structures [7]. Huber experimented with sugar currents through bottom slots and generated a series of critical densimetric Froude numbers [8]. Hocking and Yu et al. conducted similar experiments, working on two-layer saline density currents through bottom slots [9, 10]. Shammaa and Zhu studied the flow field induced by the bottom jet and the mean flow in a two-layer flume with a temperature-control curtain [11]. However, these experiments focus on velocity field or turbulent jet, not on the withdrawn water temperature. The rules of heat transfer may not necessarily be followed; thus, the similarity theory they adopted is significantly different from ours. Information on similarity theory of withdrawn water temperature model remains lacking for selective withdrawal from a thermal stratified reservoir.

In China, research on temperature similarity theory was conducted previously in the field of thermal discharge [12]. Heat exchange at the interface of water and gas was considered. Temperature change was decided by buoyant jet and turbulent mixing [13], which was not similar to the withdrawn water temperature model. With regard to studying underwater glider in ocean thermocline [14], although they mentioned the temperature stratified in ocean, they were concerned more with motion and dynamic similarity not with the temperature. Thus, similarity theory is necessary for a model test in the study of withdrawn water temperature using selective withdrawal from a thermal stratified reservoir.

This paper presents similarity theory of withdrawn water temperature model test. The flow feature of withdrawn water temperature model will first be reviewed. The modification of the governing equations and the precondition of the experiment similarity will then be presented. In the following section, we propose the primary theory of temperature distribution conversion, and the conversion of the withdrawn water temperature is suggested subsequently. Such is followed by a verification of the similarity theory.

With the similarity theory we proposed, experiments of withdrawn water temperature could be conducted no matter whether the basic temperatures of the model were consistent with that of the prototype or not. And this would greatly improve the efficiency of the experiment.

## 2. Theoretical Analysis 

### 2.1. Flow Features of Withdrawn Water Temperature Model

**Figure 1:**
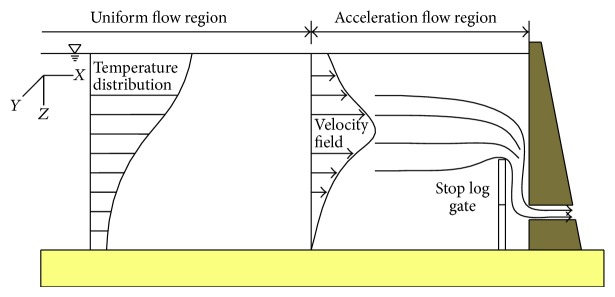
Flow regions in the reservoir.

In a thermal stratified reservoir, water in the middle of the reservoir moves slowly and horizontally [15]. In the region near the intake, the flow began to accelerate and changed its flow direction to the intake. The reservoir was divided into two parts according to flow feature, namely, the uniform and the acceleration flow regions (Figure 1).

In the uniform flow region, the water flowed very slowly with a velocity direction only along the *X* direction. According to heat flux conservation, the heat flux in unit time and area perpendicular to the *X* direction could be calculated by the equation *q*_*xi*_ = *c*_*p*_ *ρu*_*xi*_ *T*_*i*_. The heat flux was in the *X* direction and the value was unchanged; so heat was transferred along thermal conduction caused by the horizontal flow.

In the acceleration flow region, water near the intake converged, and both the flow rate and the direction significantly changed. These changes led to the redistribution of the temperature; thus heat conduction and convection in this region must be considered.

**Figure 2:**
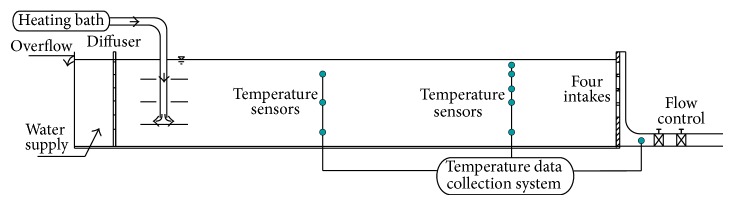
Schematics of selective withdrawal using stop log gates.

To verify the similarity theory, we conducted an experiment to study the withdrawn water temperature [16]. The experiment was designed based on the idea of generating a continuous temperature distribution with the same flow direction in the entire flume. A rectangular flume that was 20 m long, 2 m wide, and 0.8 m high with a horizontal bottom was selected (Figure 2). The flume was fixed on a water feeding tank. The outflow was measured at the end of the flume. The water level in the reservoir was controlled by a weir at the far end of the flume, and the overflow water returned to the feeding tank. Aside from the flume, the experiment system contained a heating control unit, a stratifying unit, a pipeline system, and a data collecting unit. The experimental data were collected and recorded synchronously using a data collection instrument within 5 min. The stop log gates were placed on the vertical direction, 10 cm in width. Compared with the entire flume we utilized in our experiment, the intake was so small that the boundary effect was no more than 5% and was negligible. Data were collected in a short period, so heat exchange at the boundary was also not considered.

### 2.2. Modification of Governing Equations Using Boussinesq Approximation

For the motion of the viscous liquid considering temperature distribution, the governing equations contained the equation for continuity, motion (N-S), and heat:

∗∂ρ∂t+∇·ρu0,

(1) dudt=g−1ρ∇p+v∇2u,

(2) dTdt=K∇2+Jρcp,

where *ρ*  is density of the water, *u* is velocity, m/s, *p* is pressure, Pa, *g* is gravitational acceleration, *v* is kinematical viscosity coefficient, *T* is water temperature, Boussinesq approximation, *K* is thermal diffusivity, and *J*/*ρc*_*p*_ is heat source.

For continuity equation, change in density likewise changes the expression:

∗∂ρ∂t+∇·ρu0,

(3) ∂ρ0+Δρ∂t+∇·ρ0+Δρu=0,

(4) ∂1+Δρ/ρ0ρ0∂t+∇·1+Δρρ0ρ0u=0.

Boussinesq approximation is based on the concept that some flows indicate minimal variation in temperature, but the buoyancy drives the motion. The density of this kind of flow minimally varies (i.e., *ρ*_*T*=4°C_ = 999.972 kg/m^3^, *ρ*_*T*=50°C_ = 988.030 kg/m^3^). Thus the variation in density is far less than 1 and could be negligible except in buoyancy. When the Boussinesq approximation was adopted, the expression would be the same as ∗.

The Boussinesq approximation was also applied to simplify motion equation.

We defined

(5) ρρ0+Δρ,p=p0+Δp,ρ0g=Δp0.

Thus, motion equation is shown as follows:

(6) ρ0+Δρdudt=ρ0+Δρg−∇p0+Δp+ρ0+Δρv∇2u,ρ0+Δρdudt=Δρg−∇Δp+ρ0+Δρv∇2u,1+Δρρ0dudt=Δρρ0g−1ρ0∇Δp+1+Δρρ0v∇2u,dudt=Δρρ0g−1ρ0∇Δp+v∇2u,dudt=Δρρ0g−1ρ0∇p−p0+v∇2u,dudt=Δρρ0g−1ρ0∇p+1ρ0ρ0g+v∇2u,dudt=g−1ρ0∇p+v∇2u+Δρρ0g.

The relationship between water density and temperature could be expressed as Δ*ρ* = −*αρ*_0_Δ*T*, where *α* is the variation of the density when temperature is changed by 1°C. Then, we obtained

$$\begin{array}{cc} \left(**\right) & \frac{du}{dt}=g-\frac{1}{\rho_{0}}\nabla p+v\nabla^{2}u-g\alpha \Delta T. \end{array}$$

By comparing (1) and ∗∗ via Boussinesq approximation, the effect of temperature is shown in the term −*gα*Δ*T*; such parameter was determined by the temperature and the physical property of water.

With regard to heat equation, in the entire experiment, heat exchange at the boundary was too minimal and, thus, was not considered. Heat equation was then expressed as *dT*/*dt* = *K*∇^2^.

Therefore, the governing equations of water temperature model are as follows:

∗∂ρ∂t+∇·ρu0,

∗∗dudt=g−1ρ0∇p+v∇2u−gαΔT,

(7) dTdt=K∇2.

## 3. Derivation and Discussion

### 3.1. Precondition of Experiment Similarity

When the model was similar to the prototype, some similar parameters must satisfy the following rules: *l*_*H*_/*l*_*M*_ = *λ*_*l*_, *u*_*H*_/*u*_*M*_ = *λ*_*u*_, *p*_*H*_/*p*_*M*_ = *λ*_*p*_, *ρ*_*H*_/*ρ*_*M*_ = *λ*_*ρ*_, *v*_*H*_/*v*_*M*_ = *λ*_*v*_, *α*_*H*_/*α*_*M*_ = *λ*_*α*_, *g*_*H*_/*g*_*M*_ = *λ*_*g*_, and Δ*T*_*H*_/Δ*T*_*M*_ = *λ*_Δ*T*_.

With these rules, the motion equation of the prototype is expressed as

(8) λu2λluM·∇uM=λggM−λpλρλl1ρM∇pM+λvλuλl2vM∇2uM−λgλαλΔTgMαMΔTM,

(9) uM·∇uM=λgλlλu2gM−λpλρλu21ρM∇pM+λvλuλlvM∇2uM−λgλαλΔTλlλu2gMαMΔTM.

The motion equation of model equation (9) was similar to ∗∗ of the prototype. Therefore, the coefficient of each term of the equation is equal to 1. Consider

(10) λgλlλu21⟹Fr=uHgHlH=uMgMlM,

(11) λpλρλu21⟹Eu=pHρHuH2=pMρMuM2,

(12) λvλuλl1⟹Re=uHlHvH=uMlMvM,

(13) λgλαλΔTλlλu21⟹Fd=uHΔρH/ρHgHlH=uMΔρM/ρMgMlM.

With regard to heat equation via the same method, we obtained

(14) $$\begin{array}{c} \frac{\lambda_{u}\lambda_{l}}{\lambda_{K}}=1⟹P_{e}=\frac{u_{H}l_{H}}{K_{H}}=\frac{u_{M}l_{M}}{K_{M}}. \end{array}$$

Therefore, the similarity criterion of the water temperature model should contain the Froude number (*F*_*r*_), Euler number (*E*_*u*_), Reynolds number (*R*_*e*_), density Froude number (*F*_*d*_), and Peclet number (*P*_*e*_). However in the model test, all similarity criteria could not be simultaneously satisfied; hence, simplification was necessary. *E*_*u*_ indicated the relative relation between pressure and inertia force. In the experiment, the flow was mainly caused by gravity, so *E*_*u*_ was not considered. Given that the intake was minimal compared with the entire flume, *R*_*e*_, which reflected the viscosity effect of the water, could similarly be omitted.

The relation between gravity and velocity is described by *F*_*r*_, which ensures the similarity of the motion. *F*_*d*_ shows the relation between momentum flux and the difference between gravity and buoyancy. These two parameters are the basic factors of the model and should be strictly followed. When *F*_*r*_ of the model was similar to that of the prototype, (13) would change to *λ*_*α*_ *λ*_Δ*T*_ = 1. For the model utilizing water as its liquid medium, *λ*_*α*_ is equal to 1 when the fundamental temperatures of the prototype and experiment are similar. In this condition, *λ*_Δ*T*_ is equal to 1 and thus indicated that the temperature difference between layers in the model must be the same as that in the prototype. Temperature distribution in the model should be the same as that of the prototype.

If we are concerned only with hydrodynamics in the thermal stratified reservoir, *F*_*r*_ and *F*_*d*_ could meet the requirement. However, with the withdrawn water temperature, another parameter, *P*_*e*_, which indicated the relative amount of the heat conduction and convection, must be considered. However, in the model test using water as its liquid medium, the three parameters could not be satisfied simultaneously.

To solve this problem, we analyzed flow feature in the flume and integrated the concept of the average withdrawn water temperature. In the uniform flow region, water flowed very slowly with a velocity along the *X* direction, so heat convection between layers could be negligible. In addition, the quantity of heat conduction would be minimal if we limited the test in a short period. And in this case, *P*_*e*_ was not necessary in this region. In the acceleration flow region, the effects of heat conduction and convection were significant. However, we discovered that although the temperature distribution was not similar to the prototype for heat change in the form of heat conduction and convection, the total heat in this region was not changed because no heat moved in or out of the region. Previous research showed that selective withdrawal affects water temperature distribution near the intake [17]. Therefore, time control was crucial for the model test. In the testing stage, withdrawn water temperature measured within 10 min varied minimally; thus, we considered water in the acceleration flow region, which was not withdrawn from the intake as quantity of heat unchanged. For the water withdrawn from the reservoir, heat conduction and convection occurred within the withdrawn water. However, the average withdrawn water temperature measured downstream was equal to that in an ideal situation. With these simplifications and time control, using the same *F*_*r*_ and *F*_*d*_, the withdrawn water temperature was the same as that of the prototype.

### 3.2. Primary Theory of Temperature Distribution Conversion

During the process of experiment, we encountered a new problem, that is, the access of significant amount of low temperature water. For temperature distribution with a low basic temperature, water with room temperature must be cooled to meet the requirement. Compared with heating up the water, cooling plenty of water required immense time and money and directly led to low efficiency of the experiment. Thus, we proposed the primary theory of temperature distribution conversion. In the previous study, we obtained the decision *λ*_*α*_ *λ*_Δ*T*_ = 1, and, thus, we should follow this rule if we wanted to maintain the similarity of temperature distributions. For the experiment with water as the medium, the relationship between water density (kg/m^3^) and temperature (°C) could be described as follows: *ρ* = *F*(*T*) = 9.8(102.027692 +0.677737262 × 10^−2^ × *T* − 0.905345843 × 10^−3^ × *T* ^2^ + 0.864372185 × 10^−5^ × *T* ^3^ − 0.642266188 × 10^−7^ × *T* ^4^ +0.105164434 × 10^−12^ × *T* ^7^ − 0.104868827 × 10^−14^ × *T* ^8^). Then, = −Δ*ρ*/*ρ*_0Δ*T*_ = −*F*′(*T*)/*F*(*T*) × Δ*T* × 1 /Δ*T* = −*F*′(*T*)/(*F*(*T*) × *T*) × *T* = *k*(*T*) × *T*. When temperature was between 10°C and 75°C, which covered the temperature range of our experiment, we got *k*(*T*) = 1 × 10^−5^. Thus, *λ*_*α*_ *λ*_Δ*T*_ = 1 became *λ*_*T*_ *λ*_Δ*T*_ = 1 and thus indicated that *T*_*P*_Δ*T*_*P*_ = *T*_*M*_Δ*T*_*M*_. If *λ*_*T*_ = 1, *λ*_Δ*T*_ = 1, and the similar condition was consistent with the abovementioned conclusion. If the basic temperatures of the model and prototype were different, *λ*_*T*_ should be first identified; then we divided the prototype temperature distribution into several layers as indicated in accuracy. Temperature distribution could be calculated from the bottom to the top reservoir by the iteration of each layer using the equation *T*_*P*_Δ*T*_*P*_ = *T*_*M*_Δ*T*_*M*_.

**Figure 3:**
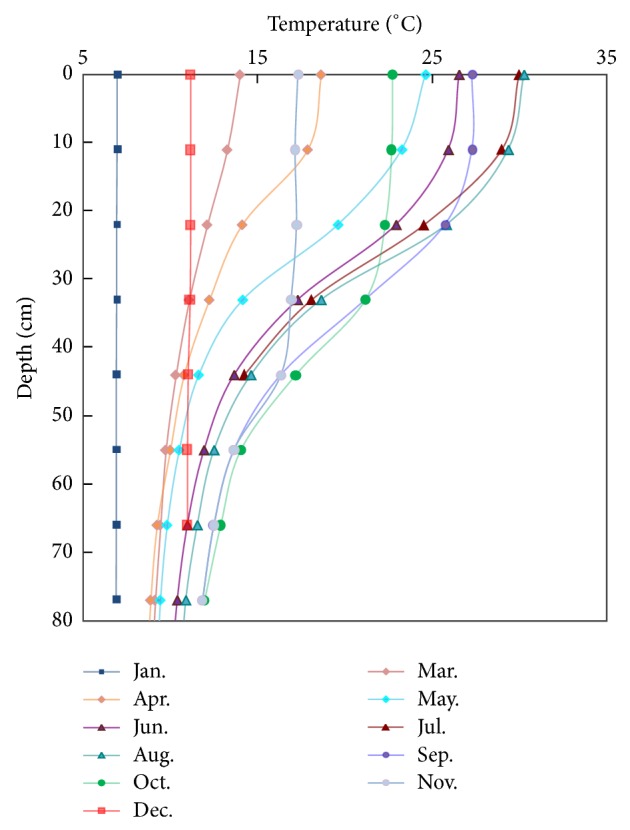
Schematics of temperature distribution.

**Figure 4:**
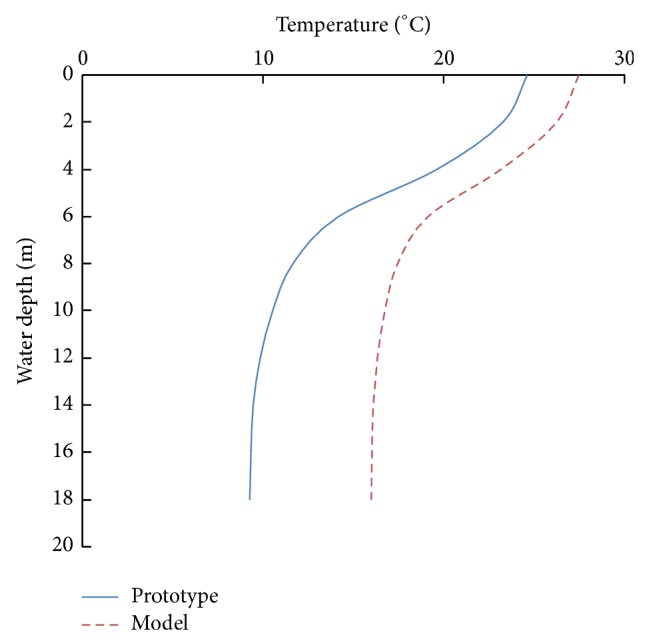
Temperature distribution of the model and the prototype.

The experiments we conducted were based on the temperature data of a thermal stratified irrigation reservoir in Jiangxi Province, China.  Figure 3 shows the observed temperature stratification in the reservoir during a typical reference year.  Figure 4 shows the temperature distribution of the prototype and calculated temperature distribution in the model with different basic temperature (using the data of May as an example).

### 3.3. Conversion of Withdrawn Water Temperature

**Figure 5:**
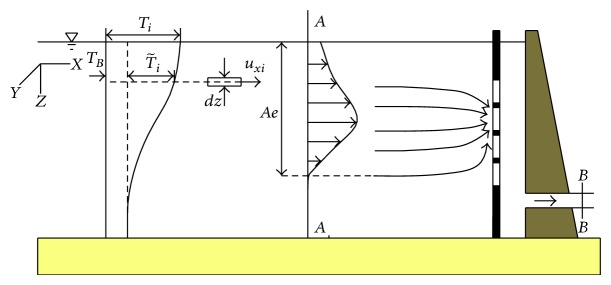
Schematic of velocity field.

The conversion relation of the withdrawn water temperature between the model and the prototype was studied. The body of water we selected was between the* A-A* and the* B-B* sections (Figure 5). There was no heat loss at the boundaries except the two sections. The heat flux at the two sections was defined as

(15) QAQB,

(16) QA=cpρ∬Aeuxi dy dzTi,

(17) QB=cρρQT−,

where *c*_*p*_ is the specific heat at constant pressure, *u*_*xi*_ is the velocity of point on* A-A* section, *T*_*i*_ is the temperature of point on* A-A* section, *Q* is the withdrawal rate, and $\overset{-}{T}$ is the withdrawn water temperature.

The temperature of water in the study region could be divided into two parts, namely, the basic and the variable $T_{i}=T_{B}+{\tilde{T}}_{i};$   thus, heat flux could be expressed as

(18) $$\begin{array}{c} Q_{A}=c_{\rho }\rho QT_{B}+c_{\rho }\rho \overset{}{\underset{Ae}{\iint }}u_{xi}\;dy\;dz{\tilde{T}}_{i}. \end{array}$$

We transformed (17) and (18) into (15). Thus, we obtained the equation

(19) $$\begin{array}{c} Q\overset{-}{T}=QT_{B}+\overset{}{\underset{Ae}{\iint }}dQ_{i}{\tilde{T}}_{i}. \end{array}$$

This equation for the prototype and the model is defined as follows:

(20) QMT−MQMTBM+∬AeMdQiMT~iM,

(21) QHT−H=QHTBH+∬AeHdQiHT~iH.

We multiplied (20) by the flow scale *λ*_*Q*_ and subtracted it from (21); thus, we obtained

(22) QHTBM−TBH+λQ∬AeMdQiMT~iM−∬AeHdQiHT~iH=QHT−M−T−H.

In the previous theoretical analysis, we discovered that if the distribution in the model was similar to that in the reservoir, ${\tilde{T}}_{iH}$ is equal to ${\tilde{T}}_{iM}$. Thus, $\lambda_{Q}\iint_{AeM}^{}dQ_{iM}{\tilde{T}}_{iM}=\iint_{AeH}^{}dQ_{iH}{\tilde{T}}_{iH}$ and the equation was transformed into

(23) $$\begin{array}{c} {\overset{-}{T}}_{H}={\overset{-}{T}}_{M}. \end{array}$$

This equation was finally a withdrawn water temperature conversion equation between the prototype and the model.

When the basic temperatures of the model and prototype were different, we used another calculation to determine the withdrawn water temperature. We divided the withdrawn water into two parts, namely, the basic and the variable temperature section. The average temperature of the variable temperature was defined as ${\overset{-}{T}}_{iM}=(\iint_{AeM}^{}dQ_{iM}{\tilde{T}}_{iM})/Q_{M}$ and ${\overset{-}{T}}_{iH}=(\iint_{AeH}^{}dQ_{iH}{\tilde{T}}_{iH})/Q_{H}$. Thus, (20) and (21) were transformed into

(24) T−MTBM+T−iM,T−H=TBH+T−iH.

The two equations also indicated that the temperature of withdrawn water was equal to that of a certain point in the reservoir. The motion of water, as well as the temperature distribution, in the model was similar to that in the prototype to a certain degree (Figure 4), especially in the middle and top reservoir. We considered that a certain point in the model and prototype contained similar elevation. Thus, we could use the equation ${\overset{-}{T}}_{H}={\overset{-}{T}}_{M}\Delta {\overset{-}{T}}_{M}/\Delta {\overset{-}{T}}_{H}$ to roughly calculate the temperature (${\overset{-}{T}}_{H}$) of the withdrawn water. $\Delta {\overset{-}{T}}_{M}$ and $\Delta {\overset{-}{T}}_{H}$ could be obtained during the distribution calculation of the new temperature distribution calculation.

The accuracy of withdrawn water temperature was determined by the temperature distribution and the basic temperature difference between the model and the prototype. Minimal difference within the reservoir and the small amplitude of temperature distribution conversion would provide highly accurate results. For the conversion of temperature distribution with the difference of basic water temperature more than 5°C, some modifications of the result must be conducted and such required further study.

### 3.4. Verification of Similarity Theory

Using the flume and the instrument mentioned above, a series of experiments were conducted according to similarity theory with the temperature date of a thermal stratified reservoir in southern China.

In the preparation stage, the entire flume was divided into three layers: the bottom layer and two heating baths were filled with water pumped from the water feeding tank. According to the original temperature of the bottom water and the objective temperature stratification, parameters were set to the heating bath, and water was automatically heated up to a certain temperature. The hot water was then poured into the flume through a special device. The device contained a large plane board, such that the water that poured onto the board would not disturb the previous layer. Meanwhile, the volume of the water pumped into the flume was strictly controlled, such that the water level in the flume rose by only 1 cm in 5 min. When the middle layer reached the appropriate elevation, pouring was stopped for a while to enable the recently poured water to be evenly mixed. Water of the top layer was poured into the flume using the same method. Finally, we could obtain a satisfactory temperature distribution.

**Figure 6:**
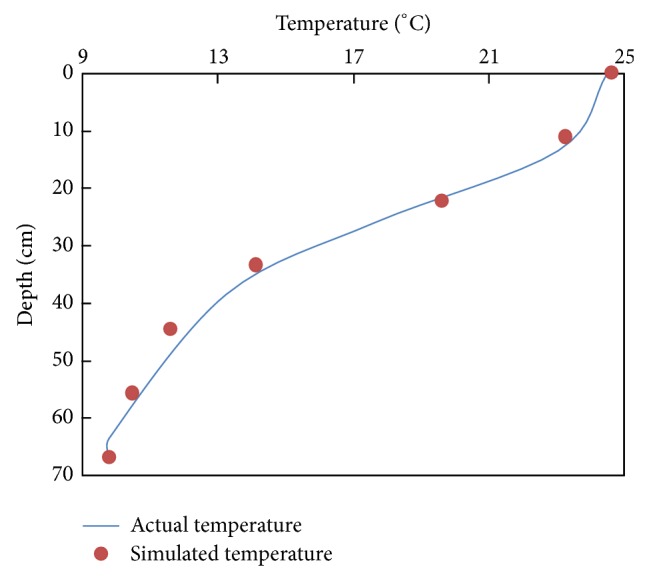
Temperature distribution in the reservoir.

**Figure 7:**
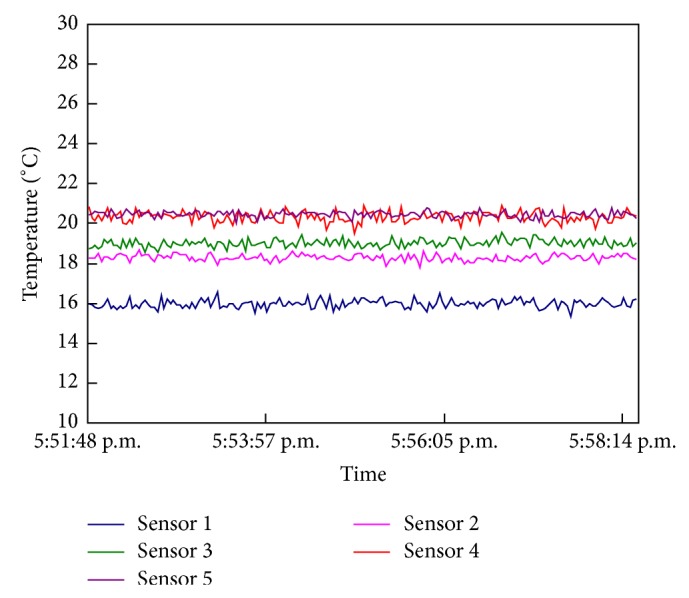
Duration curve of temperature sensors.

**Figure 8:**
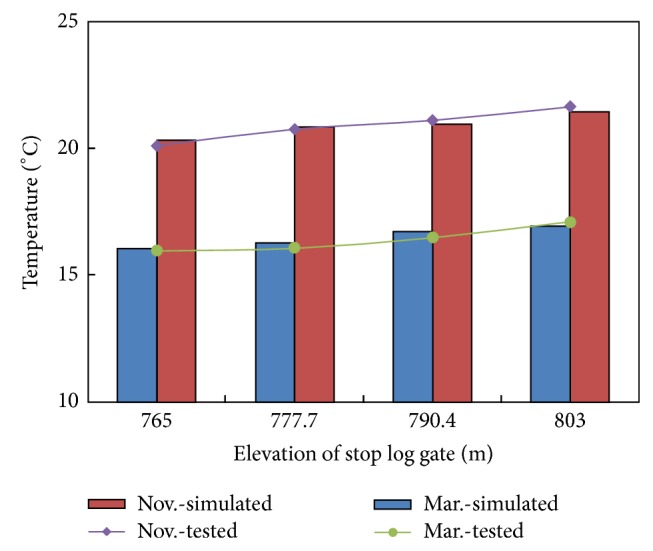
Comparison between the simulated and test values.

The simulated temperature distribution suitably matched the actual temperature distribution in the reservoir (Figure 6). The collected data showed that the temperature stratification near the intake would not change in the first 10 min (Figure 7). Thus, the data on withdrawn water temperature collected synchronously within 10 min were reliable and accurate. A comparison of withdrawn water temperature between the model test and numerical simulation (using EFDC) [18] is shown in Figure 8. We could easily find that we correspond with each other.

## 4. Conclusion

To research the withdrawn water temperature, experiment study was an essential and feasible method. Thus, similarity theory was necessary. In this paper, a similarity theory of withdrawn water temperature model was proposed with the Boussinesq approximation and some simplifications. For the experiment study, the withdrawn water temperature of the model was the same as that of the prototype under the conditions of the same *F*_*r*_, *F*_*d*_ and temperature distribution. Considering the feasibility and the efficiency of the model, we proposed a primary theory of temperature distribution conversion. Using this theory, the experiment could be conducted even with a different basic temperature of the model. The conversion of temperatures of withdrawn water was likewise suggested. When the basic temperature of the model was the same as the prototype, the temperature of the withdrawn water could be calculated as ${\overset{-}{T}}_{H}={\overset{-}{T}}_{M}$. When the basic temperatures of the model and prototype are different, it would transform into ${\overset{-}{T}}_{H}={\overset{-}{T}}_{M}\Delta {\overset{-}{T}}_{M}/\Delta {\overset{-}{T}}_{H}$. The comparison between the tested value and simulated value verified the feasibility of the similarity theory.
